# Frailty Significantly Associated with a Risk for Mid-term Outcomes in Elderly Chronic Coronary Syndrome Patients: a Prospective Study

**DOI:** 10.21470/1678-9741-2019-0484

**Published:** 2020

**Authors:** Caglar Ozmen, Ali Deniz, İmam Günay, İlker Ünal, Aziz Inan Celik, Çağlar Emre Çağlıyan, Onur Sinan Deveci, Mesut Demir, Mehmet Kanadaşı, Ayhan Usal

**Affiliations:** 1 Department of Cardiology, Faculty of Medicine, Cukurova University, Adana, Turkey.; 2 Department of Biostatistics, Faculty of Medicine, Cukurova University, Adana, Turkey.; 3 Department of Cardiology, Gebze Fatih State Hospital, Kocaeli, Turkey.

**Keywords:** Frailty, Frail Elderly, Risk Factors, Confidence Intervals, Multivariate Analysis, Prognosis, Canada, Aging, Death, Heart

## Abstract

**Introduction:**

Frailty is a condition of elderly characterized by increased vulnerability to stressful events. Frail patients are more likely to have adverse events. The purposes of this study were to define frailty in patients aged ≥ 70 years with chronic coronary syndrome (CCS) and to evaluate mortality and prognostic significance of frailty in these patients.

**Methods:**

We included 99 patients, ≥ 70 years old (mean age 74±5.3 years), with diagnosis of CCS. They were followed-up for up to 12 months. The frailty score was evaluated according to the Canadian Study of Health and Aging (CSHA). All patients were divided as frail or non-frail. The groups were compared for their characteristics and clinical outcomes.

**Results:**

Fifty patients were classified as frail, and 49 patients as non-frail. The 12-month Major Adverse Cardiac Events (MACE) rate was 69.4% in frail patients and 20% in non-frail patients. Frailty increases the risk for MACE as much as 3.48 times. Two patients died in the non-frail group and 11 patients died in the frail group. Frailty increases the risk for death as much as 6.05 times. When we compared the aforementioned risk factors by multivariate analysis, higher CSHA frailty score was associated with increased MACE and death (relative risk [RR] = 22.94, 95% confidence interval [CI] 3.33-158.19, *P*=0.001, for MACE; RR = 7.41, 95% CI 1.44-38.03, *P*=0.016, for death).

**Conclusion:**

Being a frail elderly CCS patient is associated with worse outcomes. Therefore, frailty score should be evaluated for elderly CCS patients as a prognostic marker.

**Table:** 

Abbreviations, acronyms & symbols				
**ACEI**	**= Angiotensin-converting enzyme inhibitor**	** **	**CVD**	**= Cardiovascular diseases**
**ACS**	**= Acute coronary syndrome**		**EF**	**= Ejection fraction**
**ARB**	**= Angiotensin II receptor blockers**		**Hb**	**= Hemoglobin**
**ASA**	**= Acetylsalicylic acid**		**HbA1c**	**= Glycated hemoglobin**
**BMI**	**= Body mass index**		**HDL**	**= High-density lipoprotein**
**BNP**	**= B-type natriuretic peptide**		**HR**	**= Hazard ratio**
**Ca**	**= Calcium**		**K**	**= Potassium**
**CABG**	**= Coronary artery bypass grafting**		**LDL**	**= Low-density lipoprotein**
**CAD**	**= Coronary artery disease**		**MACE**	**= Major Adverse Cardiac Events**
**CCS**	**= Chronic coronary syndromes**		**Mg**	**= Magnesium**
**CI**	**= Confidence interval**		**MI**	**= Myocardial infarction**
**COPD**	**= Chronic obstructive pulmonary disease**		**Na**	**= Sodium**
**CRP**	**= C-reactive protein**		**PCI**	**= Percutaneous coronary intervention**
**CSHA**	**= Canadian Study of Health and Aging**		**RR**	**= Relative risks**

## INTRODUCTION

Cardiovascular diseases (CVD) are the number one cause of death in the world^[1]^. With a prolonged life expectancy and the development of interventional treatment for these diseases, new problems, such as geriatric syndromes, are evolving. Several studies have reported the mortality rate of Major Adverse Cardiac Events (MACE) between the ages of 60-80 years and > 80 years as 4.1% and 11.5%, respectively, which is far higher than of people < 60 years old, who have a 1% mortality rate^[2,3]^.

Frailty is a physiological decline of bodily functions that can occur in every organ system. It is determined by old age and is associated with comorbidities and disabilities^[2]^. Frail patients with coronary artery disease (CAD) are more likely to experience adverse events than non-frail people^[4]^. In elderly CAD patients, a longer life expectancy has led to a greater focus on improving the patients’ ability to function and their quality of life^[5]^. As a result, the concept of frailty has attracted increasing attention as a mean of identifying patients more prone to poor outcomes in coronary events^[6]^.

Although the pathways leading to frailty and CAD are involved, they both have been strongly associated with chronic low-grade inflammation^[7]^. Chronic inflammation results in the oxidation of lipoproteins and the activation of atheromatous plaques^[8]^. As frailty involves multisystem physiologic dysregulation, it is conceivable that chronic inflammation contributes to frailty through its detrimental effects on other physiologic organ systems, such as the musculoskeletal and endocrine systems, anemia, and clinical and subclinical CAD^[9,10]^. In addition to sharing causal pathways, CAD can contribute to the development of frailty^[11]^.

Less is known about frailty and chronic coronary syndromes (CCS); and frailty is rarely used in the assessment of cardiovascular treatment and prognosis. Frailty reflects biological rather than chronological age, and this may explain why there is significant heterogeneity in clinical outcomes in the elderly patient population. The purposes of this study were to define frailty in patients aged ≥ 70 years with CCS and to evaluate the mortality and prognostic significance of frailty in these patients.

## METHODS

This prospective study was conducted in accordance with the Declaration of Helsinki and the Good Clinical Practice Guidelines. Our study was approved by the local ethics committee issued by the University of Cukurova, number 90/15. All patients enrolled in the study signed written informed consent. Between January 2018 and August 2018, we prospectively enrolled 99 consecutive patients, aged ≥ 70 years, with diagnosis of CCS, and who underwent elective coronary angiography with or without percutaneous coronary intervention (PCI) at Cukurova University Hospital, Cardiology Department. The exclusion criteria were as follows: (1) if the patient was not willing to participate; (2) if the patient was diagnosed with acute coronary syndrome (ACS).

This is a prospective study addressing a Canadian Study of Health and Aging (CSHA) frailty scoring system that, to the best of our knowledge, has not been used previously to predict the risk of mid-term outcomes for CCS patients. The clinical and laboratory data and echocardiography were collected and recorded. The patients were divided into two groups, frail and non-frail patients, according to the CSHA frailty scoring system. The frailty scores of the patients were also evaluated by this system^[12]^. The CSHA frailty scale is a seven-point scale that determines frailty scores with a good prognostic value, depending on clinical judgment. The patients are frail if the score is ≥ 5.

The impact of comorbidities on risk of CAD was quantified by a CAD-specific index^[13]^. In this scoring system, each separate significant comorbidity (hypertension, current smoker, history of a cerebrovascular event, chronic obstructive pulmonary disease, metastatic tumor, renal disease, peripheral vascular disease, tumor, and diabetes mellitus with complications) has a coefficient, and the final score is calculated by the sum of the factors. The patients are at a low to moderate risk if the score is 0-3 and at high risk if it is ≥ 4.

PCI was performed according to the physicians’ decision and preference depending on the patients’ clinical and angiographic statuses. The severity and the burden of coronary atherosclerosis were determined based on the Gensini score^[14]^. This score is associated with short- and long-term cardiovascular risk and considers artery morphology, coronary anatomy, and the severity of stenosis in lesions. Patients with Gensini scores ≥ 20 were defined as having equivalent to severe CAD, which meant they had approximately 70% or more stenosis lesions in the proximal left anterior descending artery^[15]^. All the scoring systems were evaluated by two cardiologists separately, and the final scores were recorded upon agreement.

Patients were followed-up for up to 12 months after the study. The patients without admission during the study’s follow-up period were called on the phone, and the relevant data were gathered by telephone interviews. The primary outcome was all-cause mortality. The secondary outcomes were composite of death, stroke/transient ischemic attack, major bleeding, and recurrent cardiovascular events during the12-month follow-up. The clinical data, history of hospitalization, major bleeding, myocardial infarction (MI), stroke, and death were recorded during each visit or during hospitalization. Bleeding was considered major if at least one of the following was present: blood transfusion, intracranial or retroperitoneal bleeding, a hemoglobin decrease of > 3 g/dL with definite cause, or a hemoglobin decrease of > 4 g/dL without apparent source^[16]^. Stroke and transient ischemic attack were defined according to a consensus statement^[17]^.

### Statistical Analysis

For each continuous variable, normality was checked by Kolmogorov-Smirnov and Shapiro-Wilk tests and by histograms. Comparisons between groups were applied using the Student’s t-test for normally distributed data, and the Mann-Whitney U test was used for the data not normally distributed. All the categorical variables were analyzed by Pearson's chi-squared test or Fisher’s exact test, as appropriate. Logistic regression was used to determine the risk factors for the presence of MACE and mortality. The data were presented at different survival rates of both the frail and non-frail patients and the Gensini low/high score patients, which were described in the Kaplan-Meier curve using the log-rank test. The Kappa statistic was used to analyze the correlation between the patients’ frailty status (absent/present), CAD-specific index (high/low), and Gensini score (high/low). Cox regression was used to calculate hazard ratios (HR) for MACE and death. The IBM SPSS Statistics Version 20.0 package program was used for the statistical analysis of the data. The results were presented as relative risks (RR) and their 95% confidence intervals (CI), as mean±standard deviation and as n (%). A *P*-value < 0.05 was considered significant.

## RESULTS

We included 99 consecutive patients, aged ≥ 70 years, and diagnosed with CCS. The mean follow-up time was 10±2 months. Their mean age was 74±5.3 years. Among them, 64 (64.7%) were between 70-79 years old and 35 (35.3%) were older (≥ 80 years). Fifty patients (50.5%) were classified as frail and 49 patients (49.5%) were non-frail. Fifty-five patients (55.6%) encountered at least one outcome (major bleeding, MI, stroke, re-hospitalization, or death) and 13 patients (13.1%) died during the follow-up period. Regarding comorbidity, six patients had malignancy, and two of them died during the follow-up period. In our study, 22 patients were revascularized. Of these patients, 19 (86.4%) had a primary outcome event, but none died within the first month of inclusion. Six (27.2%) of these 22 patients died during the 12-month follow-up.

When comparing frail and non-frail patients, determined as such according to the CSHA, the groups were similar with respect to age, diabetes mellitus, chronic renal disease, anemia, distribution of gender, body mass index, blood pressure, heart rate, laboratory data (except for calcium), and low-density lipoprotein (LDL) levels (Table 1). Calcium and LDL levels were found to be higher in frail patients (calcium level: 9.0±0.9 mg/dL in frails, 8.3±1.9 in non-frails, *P*=0.045; LDL level: 137.0±48.0 mg/dl in frails, 106.5±32.2 in non-frails, *P*<0.001). There were lower uses of angiotensin-converting enzyme inhibitors and angiotensin receptor blockers (50.0% *vs*. 77.6%, *P*=0.006) and higher uses of acetylsalicylic acid (86% *vs*. 67.3%, *P*=0.034) in frail patients compared to non-frail patients. Ejection fraction (EF) values were similar between the groups (EF: 56.2±10.0% in non-frails, 53.0±12.4% in frails, *P*=0.114). Frail patients presented a greater burden of comorbidities, including higher rates of a PCI history and malignancy (38.0% *vs*. 12.2% for PCI history; 12.0% *vs*. 0% for malignancy, *P*=0.005 and *P*=0.027, respectively). Revascularization ratio (PCI or coronary artery bypass grafting) was found to be higher in frail patients (38% in frails, 6.1% in non-frails, *P*<0.001). They also had a higher CAD-specific index score and Gensini score (10.2±2.7 *vs*. 2.95±2.1, *P*=0.027 for CAD-specific index score; 71.42±21 *vs*. 23.73±13, *P*<0.001 for Gensini score) (Table 1).

**Table 1:** Comparison of the the baseline characteristics.

	Non-frail patients (n=49)	Frail patients (n=50)	*P*-value
Gender (%) (male)	63.3	72.0	0.395
Age (year)	74.7±7.6	77.8±9.4	0.071
Hypertension (%)	67.3	74.0	0.513
Diabetes mellitus (%)	32.7	48.0	0.153
Current smoking (%)	28.6	34.0	0.560
CABG history (%)	14.3	20.0	0.451
COPD (%)	18.4	16.0	0.795
Chronic renal disease (%)	6.1	4.0	0.678
Stroke history (%)	10.2	8.0	0.741
PCI history (%)	12.2	38.0	0.005
Dementia (%)	10.2	14.0	0.563
Peptic ulcer (%)	20.4	10.0	0.171
Anemia (%)	22.4	14.0	0.276
Osteoporosis (%)	20.4	16.0	0.570
Married/single (%)	85.7/14.3	92.0/8.0	0.357
Malignancy history (%)	0	12.0	0.027
BMI (kg/m^2^)	28.9±7.4	28.8±10.1	0.945
Heart rate (bpm)	77.3±8.9	80.6±13.8	0.163
Systolic blood pressure (mmHg)	129.2±15.6	134.5±15.2	0.094
Diastolic blood pressure (mmHg)	77.8±6.5	76.8±7.7	0.487
Hb (mg/dl)	12.2±1.8	12.4±1.6	0.865
Creatinine (mg/dl)	0.79±0.18	0.88±0.27	0.056
Uric acid (mg/dl)	4.8±1.7	4.5±1.9	0.479
BNP	544.5±1044	601.3±1138	0.797
LDL	106.5±32.2	137.0±48.0	< 0.001
HDL	43.9±12.4	41.6±8.3	0.284
CRP	7.8±11.9	5.2±8.1	0.201
Na	139.2±3.2	138.1±3.3	0.114
K	4.2±0.4	4.3±0.4	0.092
Ca (mg/dL)	8.3±1.9	9.0±0.9	0.045
Mg	1.9±0.7	2.0±0.6	0.522
HbA1c	3.5±3.5	2.4±3.5	0.134
Albumin (gr/dl)	2.9±0.2	3.1±0.2	0.338
Ejection fraction (%)	56.2±10.0	53.0±12.4	0.114
ASA (%)	67.3	86.0	0.034
Clopidogrel (%)	18.4	32.0	0.165
ASA+clopidogrel (%)	14.3	30.0	0.090
Ticagrelor (%)	2.0	10.0	0.204
Beta blocker (%)	46.9	62.0	0.160
ACEI or ARB (%)	77.6	50.0	0.006
Statin (%)	32.7	50.0	0.103
Calcium antagonist (%)	32.7	26.0	0.513
Nitrate (%)	20.4	30.0	0.272
Revascularization (PCI or CABG) (%)	6.1	38.0	< 0.001
CAD-specific index score	2.95±2.1	10.2±2.7	0.027
Gensini score	23.73±13	71.42±21	< 0.001

ACEI=angiotensin-converting-enzyme inhibitor; ARB=angiotensin II receptor blockers; ASA=acetylsalicylic acid; BMI=body mass index; BNP=B-type natriuretic peptide; Ca=calcium; CABG=coronary artery bypass grafting; CAD=coronary artery disease; COPD=chronic obstructive pulmonary disease; CRP=C-reactive protein; Hb=hemoglobin; HbA1c=glycated hemoglobin; HDL=high-density lipoprotein; K=potassium; LDL=low-density lipoprotein; Mg=magnesium; Na=sodium; PCI=percutaneous coronary intervention

**Fig. 1:**
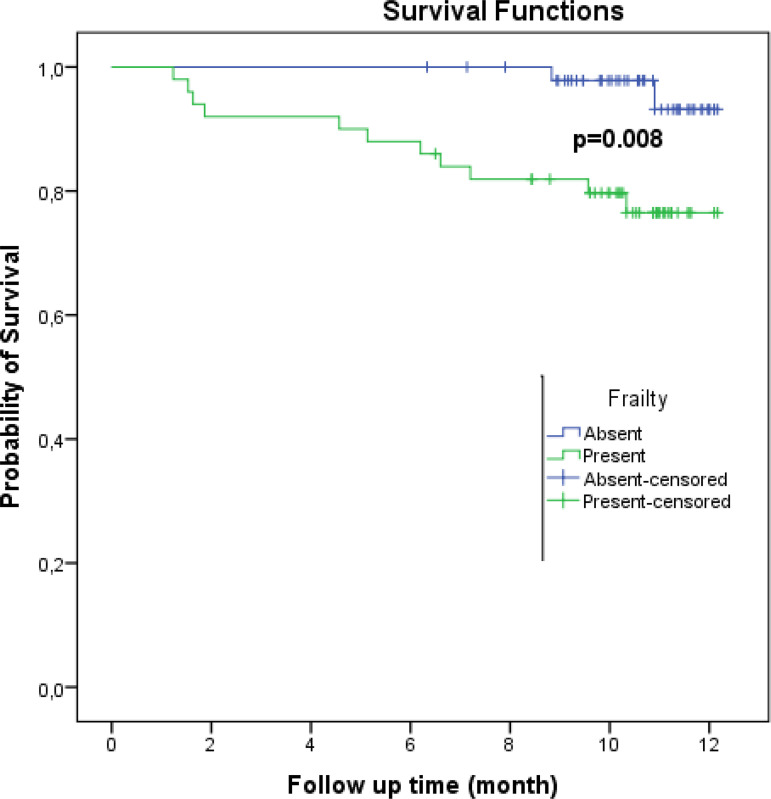
Kaplan-Meier survival curves for the frailty. Survival probabilities and their 95% confidence ıntervals in the time “12 months” are 0.93 (0.83-1.00) for non-frail patients and 0.77 (0.64-0.90) for frail patients.

Within the follow-up period, 13 patients (13.1%) died. Two patients (4.1%) in the non-frail group died (including two sudden deaths and one MI) and 11 patients (22.0%) died in the frail group (including two sudden deaths, two worsening heart failures, two neoplasms, two lung infections, and multiple organ failures). Patients who died also had high frailty score and Gensini score (frailty score: 5.5±1.3 *vs*. 4.4±1.7, *P*=0.026; Gensini score: 74.9±35.9 *vs*. 43.7±37.2, *P*=0.006). There were significantly more smoking habits found in the group with deaths (61.5% *vs*. 26.7%, *P*=0.021). The albumin levels were found to be significantly lower in both the group with deaths and MACE (3.3±0.6 *vs*. 3.7±0.5 gr/dl, *P*=0.014, for MACE; and 3.1±0.7 *vs*. 3.7±0.5 gr/dl, *P*=0.014, for death) (Table 2). Figure 1 shows the Kaplan-Meier survival estimates according to frailty status. In our study, the 12-month survival rate was 77% in frail patients and 93% in non-frail patients. Frailty was independently associated with a risk for death by multiple regression analyses (RR = 7.41, 95% CI 1.44-38.03, *P*=0.016). The effect of frailty on death can be seen based on the crude HR, which was 6.05 (95% CI 1.34-27.31). In our study, the 12-month survival rate was 97.3% in low-moderate CAD-specific index patients and 80.6% in high CAD-specific index patients. The 12-month survival rate was 95.7% in patients with a low Gensini score and 79.2% in patients with a high Gensini score.

**Table 2:** Comparison of the the baseline characteristics, laboratory, and frailty score according to the end points.

	MACE			Death		
	Present	Absent	P-value	Present	Absent	*P*-value
Gender (%) (female/male)	40.0/60.0	22.7/77.3	0.077	46.2/53.8	30.2/69.8	0.340
Age (year)	76.9±9.0	75.5±8.2	0.457	76.2±9.8	76.3±8.5	0.967
Hypertension (%)	65.5	77.3	0.199	84.6	68.6	0.335
Diabetes mellitus (%)	50.9	27.3	0.017	53.8	38.4	0.289
Current smoking (%)	34.5	27.3	0.438	61.5	26.7	0.021
CABG history (%)	18.2	15.9	0.766	30.8	15.1	0.229
COPD (%)	14.5	20.5	0.439	15.5	17.4	1.000
Chronic renal disease (%)	3.6	6.8	0.653	15.4	3.5	0.127
Stroke history (%)	10.9	6.8	0.727	15.2	8.1	0.336
Malignancy history (%)	10.9	0	0.032	7.7	5.8	0.580
BMI (kg/m²)	30.3±10.0	27.0±6.6	0.069	26.0±5.4	27.6±6.1	0.677
Heart rate (bpm)	79.0±13.1	79.0±9.6	0.985	77.5±18.6	79.2±10.3	0.752
Systolic blood pressure (mmHg)	136.8±13.6	125.7±15.6	<0.001	133.2±16.0	131.7±15.5	0.748
Diastolic blood pressure (mmHg)	77.6±7.8	76.8±6.0	0.554	77.3±8.8	77.2±6.8	0.994
Hb (mg/dl)	12.3±1.9	12.4±1.7	0.879	11.6±1.9	12.4±1.6	0.280
Creatinine (mg/dl)	0.8±0.2	0.8±0.2	0.341	0.78±0.2	0.84±0.2	0.347
Uric acid (mg/dl)	7.1±1.9	6.7±1.7	0.401	7.2±1.7	6.7±1.7	0.455
BNP (pg/mL)	575.4±1074	570.3±1116	0.982	442.0±930.5	593.0±1113.0	0.983
LDL (mg/dl)	136.8±46.2	103.2±31.6	< 0.001	122.0±45.7	121.8±43.5	0.989
HDL (mg/dl)	41.8±8.8	43.8±12.3	0.353	43.08±9.9	42.7±10.7	0.909
CRP (mg/L)	6.1±9.0	6.8±11.6	0.750	4.4±4.4	10.8±6.7	0.697
Na (mEq/L)	138.8±3.5	138.4±2.9	0.574	139.0±3.4	138.5±3.2	0.606
K (mEq/L)	4.3±0.4	4.1±0.4	0.003	4.2±0.4	4.2±0.5	0.854
Ca (mg/dL)	8.6±1.3	8.6±1.8	0.896	9.1±0.7	8.6±1.6	0.252
Mg (mEq/L)	1.9±0.7	2.0±0.6	0.594	2.1±0.4	1.9±0.7	0.224
HbA1c (% mmol/mol)	2.3±3.5	3.6±3.3	0.080	3.0±1.9	3.6±3.1	0.351
Albumin (gr/dl)	3.3±0.6	3.7±0.5	0.014	3.1±0.7	3.7±0.5	0.014
Ejection fraction (%)	52.2±11.9	57.0±10.2	0.035	52.8±10.6	54.6±11.6	0.612
ASA (%)	81.8	70.5	0.183	92.3	74.4	0.289
Clopidogrel (%)	27.3	22.7	0.605	38.2	23.3	0.304
ASA+clopidogrel (%)	25.5	18.2	0.387	38.5	19.8	0.156
Ticagrelor (%)	7.3	4.5	0.690	7.7	5.8	0.580
Beta blocker (%)	52.3	46.0	0.653	54.6	47.5	0.314
ACEI or ARB (%)	56.4	72.7	0.093	46.2	66.3	0.217
Statin (%)	56.3	43.5	0.266	59.5	47.2	0.322
Calcium antagonist (%)	21.8	38.6	0.078	15.4	31.4	0.335
Nitrate (%)	32.7	15.9	0.066	23.1	25.6	1.000
Frailty score (CSHA)	5.2±1.5	3.6±1.2	< 0.001	5.5±1.3	4.4±1.7	0.026
CAD-specific index score	7.3±2.5	3.1±2.8	< 0.001	6.5±2.3	5.4±3.5	0.161
Gensini score	66.4±38.5	24.4±22.1	< 0.001	74.9±35.9	43.7±37.2	0.006

ACEI=angiotensin-converting enzyme inhibitor; ARB=angiotensin II receptor blockers; ASA=acetylsalicylic acid; BMI=body mass index; BNP=B-type natriuretic peptide; Ca=calcium; CABG=coronary artery bypass grafting; CAD=coronary artery disease; COPD=chronic obstructive pulmonary disease; CRP=C-reactive protein; CSHA=Canadian Study of Health and Aging; Hb=hemoglobin; HbA1c=glycated hemoglobin; HDL=high-density lipoprotein; K=potassium; LDL=low-density lipoprotein; MACE=Major Adverse Cardiac Events; Mg=magnesium; Na=sodium

**Fig. 2:**
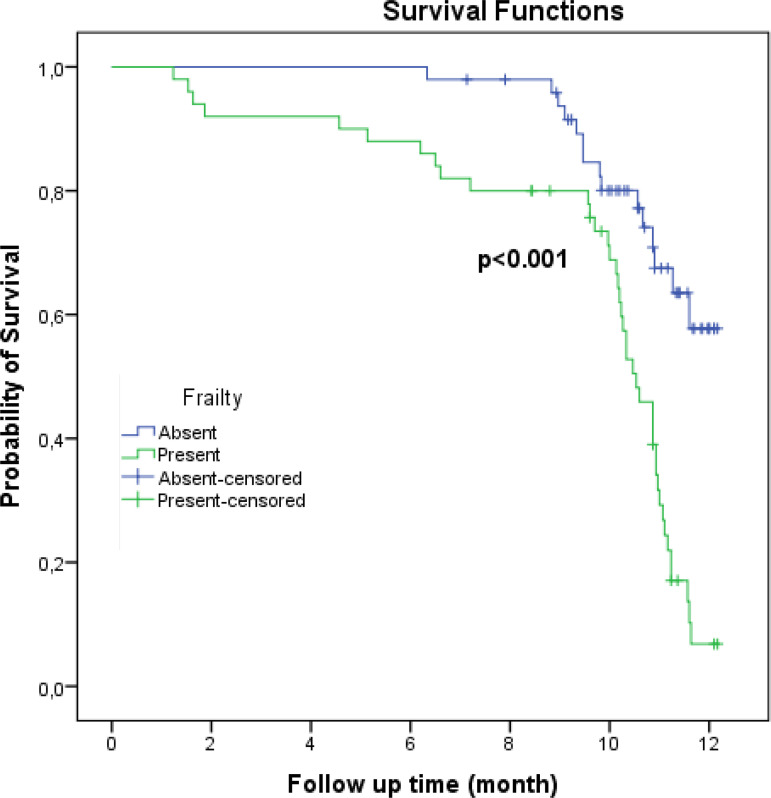
Kaplan-Meier curves of freedom from Major Adverse Cardiac Events (MACE) to one year. MACE probabilities and their 95% confidence ıntervals in the time “12 months” are 0.58 (0.40-0.76) for non-frail patients and 0.07 (0.00-0.15) for frail patients.

The overall MACE incidence was 55.6%. In our study, the 12-month MACE rate was 69.4% in frail patients and 20% in non-frail patients. The MACE incidence included 13 deaths, three restenosis, seven MI, 17 events of re-hospitalization, 10 major bleedings, and five events of a stroke. The effect of frailty on MACE can be seen based on the crude HR, which was 3.48 (95% CI 1.91-6.33). In the non-frail group, 15 patients had MACE, whereas in the frail group, 40 patients experienced MACE. Patients who experienced MACE also had high systolic blood pressure and potassium levels (136.8±13.6 *vs*. 125.7±15.6 mmHg, *P*<0.001; 4.3±0.4 *vs*. 4.1±0.4 mEq/L, *P*=0.003, respectively). The frequency of diabetes mellitus, malignancy history, LDL levels, and the CAD-specific index score were found to be significantly higher only in the group with MACE (diabetes mellitus: 50.9% *vs*. 27.3%, *P*=0.017; malignancy history: 10.9% *vs*. 0%, *P*=0.032; LDL levels: 136.8±46.2 *vs*. 103.2±31.6 mg/dl, *P*<0.001; CAD-specific index score: 7.3±2.5 *vs*. 3.1±2.8, *P*<0.001) (Table 2). The Kaplan-Meier test showed significant results (*P*<0.001, log-rank test, Figure 2). Based on the results, frail patients also experienced MACE earlier than non-frail patients (9.5 months 95% CI 8.7-10.3 *vs*. 11.3 months 95% CI 10.9-11.7).

When the association between the frailty score and CAD-specific index score was examined, a weak correlation between these parameters was found (kappa = 0.311). When the association between the frailty score and Gensini score was observed, a high correlation between these parameters was found (kappa = 0.697).

When we compared the aforementioned risk factors by a multivariate analysis, a higher CAD-specific index score was associated with a significantly higher risk for MACE (RR = 151.58, 95% CI 15.18-1513.39, *P*<0.001) (Table 3). A higher CSHA frailty score was associated with an increase in both MACE and death (RR = 22.94, 95% CI 3.33-158.19, *P*=0.001, for MACE; RR = 7.41, 95% CI 1.44-38.03, *P*=0.016, for death). A low albumin level was significantly associated with MACE for the mid-term follow-up (RR = 0.91, 95% CI 0.84-0.97, *P*=0.006). Ages > 80 years were significantly associated with MACE for the mid-term follow-up (RR = 7.57, 95% CI 1.06-53.99, *P*=0.044). A current smoking habit was significantly associated with death for the mid-term follow-up (RR = 4.68, 95% CI 1.29-16.93, *P*=0.019). High Gensini score and reduced EF (< 40%) were not associated with MACE (*P*=0.487 and *P*=0.726, respectively). No other factor was associated with the composite outcome in the adjusted analysis.

**Table 3:** Comparison of the major prognostic parameters according to the end points by multiple logistic regression analysis.

**MACE**	***P*-value**	**RR**	**95% CI for RR**	
			**Lower**	**Upper**
Albumin	0.006	0.91	0.84	0.97
Frailty (CSHA) - present	0.001	22.94	3.33	158.19
CAD-specific index score ≥ 4	<0.001	151.58	15.18	1513.39
Age ≥ 80 years	0.044	7.57	1.06	53.99
Gensini score ≥ 20	0.487	0.52	0.08	3.29
EF < 40	0.726	1.39	0.22	8.61
**Death**	***P*-value**	**RR**	**95% CI for RR**	
			**Lower**	**Upper**
Smoking (current)	0.019	4.68	1.29	16.93
Frailty (CSHA) - present	0.016	7.41	1.44	38.03
Age ≥ 80 years	0.550	0.67	0.18	2.53

CAD=coronary artery disease; CI=confidence interval; CSHA=Canadian Study of Health and Aging; EF=Ejection fraction; MACE=Major Adverse Cardiac Events; RR=relative risk

## DISCUSSION

In this study, frailty was one of the most potent risk predictors of MACE and death in CCS patients. Our prospective cohort study addresses the importance of classifying elderly CCS patients according to their frailty status to predict the development of essential health outcomes during a mid-term follow-up. In our study, 50.5% of the patients were frail, frailty was related to all-cause mortality, and 22.0% of frail patients experienced cardiovascular death over the 12 months of follow-up. To our knowledge, this is the first study demonstrating frailty as a risk factor for adverse mid-term clinical outcomes for stable elderly CAD patients.

Along with the increase of society’s life expectancy, the number of critically ill elderly patients has been increasing^[18]^. Frailty carries a relative morbidity and mortality risk of > 2 across a spectrum of ACS, CCS, and transcatheter or surgical interventions^[6]^. Our findings are valuable because frailty may reflect the age-related heterogeneity of older populations better than chronological age alone.

It is worth noting that in our study, a high risk in the CAD-specific index was associated with a higher risk for MACE in CCS patients in an adjusted analysis with the use of multiple regression. Concerning the burden of comorbidity in our study, 62.6% of the patients had high CAD-specific index scores. This represents a higher proportion than in recent studies using the same index^[13,19]^ We believe this may be because our study included more elderly patients than those recent studies. Frailty and CVD share common risk factors (*i.e*., insulin resistance, inflammation, increased age, etc.)^[20]^. When the relationship between frailty and CVD is examined, there is significant evidence that the presence of one condition can accelerate or worsen the development of the other. Frail patients with CVD, especially those undergoing invasive methods such as PCI, are more likely to have more adverse effects than people without frailty^[21]^. Therefore, identifying patients at an earlier stage, such as those with frailty, for preventive interventions can break this vicious cycle^[22]^.

Gensini scores involve an analysis of both the percentage of stenosis and the coronary artery morphology, which is associated with long-term cardiovascular outcomes^[14,23]^. In our study, frail patients had a higher Gensini score and although patients who experienced MACE also had high Gensini score, this score was not associated with MACE in the multivariate analysis.

There were 13 deaths in this study, and the mortality rate in the 12-month period was 13.1%. We did not observe any deaths in the first 30 days. Gharacholou SM et al.^[24]^ have assessed the incidence of death in patients aged 65 years over 35 months. They have found a 12% mortality rate. Upon further analysis, they have also revealed that during a three-year follow-up, frail patients had a four-fold increased risk of death. In a Canadian study by Heyland DK et al.^[25]^, a lower index on a frailty scale was independently associated with an improved survival rate for a 12-month period. We also showed frailty to be strongly associated with mortality in CCS patients. These results are compatible with the evidence demonstrating the prognostic role of frailty. Our findings demonstrate the feasibility of using the CSHA in the elderly CCS patient population. We also believe this simple-to-use clinical frailty scale should be a component for the risk evaluation of elderly CCS patients.

In our study, we found a relationship between MACE and low albumin and K levels. The inflammation and frailty relationship is complicated, as they both linearly increase with age. Frail patients are exposed to a higher presence of factors, such as disability and medical conditions, that are potent to increase the inflammatory processes. Inflammation is known for its key role in the oxidation of lipoproteins and activation of plaques in CVD^[8]^. Studies have shown that levels of albumin have inverse associations with elevated molecular and cellular inflammatory agents. In frailty, inflammation stimulates a catabolic process that helps redistribute amino acids from skeletal muscles to vital organs, which results in less amino acids available for repair and maintenance functions^[10]^.

In our study, we found an association between MACE and malignancy history. In the literature, the prevalence of frailty in older malignant patients is high. Routine assessments of frailty in older malignant patients may have a role in guiding the treatment. In our study, patients with malignant tumors (six patients) were also included. These patients were always excluded in previous studies. Therefore, our study is closer to the experiences in the real world. Failure to detect frailty potentially exposes older cancer patients to treatments from which they might not benefit and may be harmed. Moreover, as many as 82.0% of the frail patients manifested one or more severe comorbid conditions (*e.g*., severe anemia, severe renal insufficiency, malignant tumor, or severe dementia). Many of these conditions are considered potential contraindications to invasive treatments. Randomized clinical studies with very few exclusion criteria are needed to study the benefits of interventions for frail CVD patients in the future.

### Limitations

There were some limitations to this study. Although it is a prospective study, our results came from a single center with a relatively small number of patients. The study did not have enough statistical power to properly analyze how frailty influences the mid-term benefit of coronary angiography and the possible invasive treatment that can follow. Therefore, larger studies would help examine the relationship between frailty and clinical outcomes. We did not have a control group, and we have no long-term survival outcome data in our study. Our study was performed with CCS patients chosen for revascularization by treating physicians. The assessment of frailty was only done at baseline and was not repeated later to assess change. Also, the chosen way of measuring frailty is subjective and may have higher inter-rater variability than more objective measures of frailty. This study was not designed to make recommendations about routine frailty screening.

## CONCLUSION

In conclusion, our study indicates frailty is strongly associated with a risk for mid-term outcomes for elderly CCS patients. Frailty was found to be associated with increased mortality. It is essential to identify clinically related measures of biological age and their contribution to risk when the population in question is the high number of elderly patients with CAD.

**Table:** 

Authors' roles & responsibilities	
CO	Agreement to be accountable for all aspects of the work in ensuring that questions related to the accuracy or integrity of any part of the work are appropriately investigated and resolved; final approval of the version to be published
AD	Drafting the work or revising it critically for important intellectual content; final approval of the version to be published
IG	Substantial contributions to the conception or design of the work; or the acquisition, analysis, or interpretation of data for the work; final approval of the version to be published
IU	Agreement to be accountable for all aspects of the work in ensuring that questions related to the accuracy or integrity of any part of the work are appropriately investigated and resolved; final approval of the version to be published
AIC	Final approval of the version to be published
CEC	Drafting the work or revising it critically for important intellectual content; final approval of the version to be published
OSD	Agreement to be accountable for all aspects of the work in ensuring that questions related to the accuracy or integrity of any part of the work are appropriately investigated and resolved; final approval of the version to be published
MD	Final approval of the version to be published
MK	Substantial contributions to the conception or design of the work; or the acquisition, analysis, or interpretation of data for the work; final approval of the version to be published
AU	Drafting the work or revising it critically for important intellectual content; final approval of the version to be published
